# Predictors of Clinical Course and Outcomes of Acute Diverticulitis: The Role of Age and Ethnicity

**DOI:** 10.3390/medicina57111269

**Published:** 2021-11-19

**Authors:** Randa Taher, Yael Kopelman, Abdel-Rauf Zeina, Amir Mari, Fadi Abu Baker

**Affiliations:** 1 Department of Internal Medicine, Hillel Yaffe Medical Center, Affiliated to the Technion Faculty of Medicine, Haifa 38100, Israel; RandaN@hy.health.gov.il; 2 Department of Gastroenterology and Hepatology, Hillel Yaffe Medical Center, Affiliated to the Technion Faculty of Medicine, Haifa 38100, Israel; yaelk@hy.health.gov.il; 3 Department of Radiology, Hillel Yaffe Medical Center, Affiliated to the Technion Faculty of Medicine, Haifa 38100, Israel; ZeinaR@hy.health.gov.il; 4 Department of Gastroenterology, Nazareth EMMS Hospital, Affiliated with the Faculty of Medicine, Bar Illan University, Safed 13100, Israel; amir.mari@hotmail.com

**Keywords:** diverticulitis, distal colon, ethnic disparity, outcome

## Abstract

*Background and Objectives*: Acute diverticulitis (AD) is the leading and most burdensome complication of colonic diverticulosis. However, risk factors for its development and predictors of its course are still poorly defined. In this regard, the association of a young age with a complicated course and worse outcome are still controversial. Moreover, little research has addressed the effect of ethnicity on the course of AD. The current study aimed to evaluate the impact of these variables on AD’s course and outcome in the diverse and unique ethnic landscape of Israel. *Materials and Methods*: We performed a retrospective review of the charts of patients with a radiologically confirmed diagnosis of AD. Patients’ outcomes and disease course, including hospitalization duration, complications, and recurrent episodes, were documented and compared among different age and ethnic groups. Multivariate analysis was performed to identify predictors of complicated AD. *Results:* Overall, 637 patients with AD were included, the majority (95%) had distal colon AD, and almost one quarter of them were aged less than 50 years. The majority of patients in the young age (<50) group were males (69.7%). Nonetheless, the rate of recurrent episodes (35.3% vs. 37.3%, *p* = 0.19), hospitalization duration (5 ± 4.7 vs. 6 ± 3.2, *p* = 0.09) and complications rate (17.3% vs. 13.7%; *p* = 0.16) were similar for both age groups. In the ethnicity group analysis, Arab minority patients had a first episode of AD at a significantly younger age compared to their Jewish counterparts (51.8 vs. 59.4 years, *p* < 0.001). However, factors such as a complicated course (16% vs. 15%; *p* = 0.08) and relapsing episode rates (33% vs. 38%; *p* = 0.36) did not differ significantly between groups. None of the variables, including young age and ethnic group, were predictors of complicated AD course in the multivariate analysis. *Conclusion:* AD is increasingly encountered in young patients, especially in ethnic minority groups, but neither ethnicity nor young age was associated with worse outcomes.

## 1. Introduction

Diverticulosis is a prevalent gastrointestinal (GI) disorder in the Western world associated with the consumption of healthcare resources and a significant economic burden [1]. The prevalence of diverticulosis increases with age, and it is estimated that almost 70% of patients aged over 80 develop diverticulosis [2]. Even though it is relatively uncommon in the younger population, recent epidemiological reports indicate that the incidence in this age group is rising [3].

Diverticulosis is essentially considered an asymptomatic condition, frequently observed as an incidental finding in patients undergoing imaging or endoscopic evaluation for diverse indications. However, a clinical presentation with diverticular bleeding, symptomatic uncomplicated diverticular disease, acute diverticulitis, or segmental colitis associated with diverticulosis is well recognized [4].

Acute diverticulitis is defined as the inflammation or infection of a diverticulum, and considered the most common diverticular disease complication, occurring in about 10–25% of diverticulosis patients [5]. A complicated diverticulitis course refers to cases presenting with free perforation, abscesses, fistula formation, or obstruction. A complicated disease is evident in almost 20% of cases, mandating advanced medical and surgical care, as well as prolonged hospitalization, and is associated with worse outcomes [6]. Numerous heterogeneous studies investigated the predictors and risk factors associated with the development, course, and outcomes of diverticulitis with conflicting results. Several reports linked advanced age, diet, obesity, and the medication use of nonsteroidal anti-inflammatory drugs (NSAIDs) and steroids to an increased risk of diverticulitis and diverticular bleeding [7,8,9,10], while current smokers appeared to be at an increased risk of abscesses and perforated diverticulitis, compared with nonsmokers [11]. The majority of the available literature on the effect of ethnicity on diverticular disease focused mainly on the differences between diverticulosis location and clinical presentation between the Asian population and their Western counterparts [12]. The specific impact of race or ethnicity on the diverticulitis course and outcomes, however, has attracted only minor interest. In this regard, Israel is a unique country with several ethnic groups, including a Jewish majority with ethnic sub-populations and an Arab minority. Moreover, Israel annually welcomes thousands of immigrants from Europe, Russia, North Africa and Asia, as well as other regions from around the globe [13]. The impact of ethnicity in the Israeli population on various GI disorders, such as gastroesophageal reflux disease [14], inflammatory bowel disease [15], *Helicobacter pylori* infection [16], and other conditions was previously outlined by different groups and contributed to improved patient management.

In our current study, we aimed to evaluate the effect of ethnicity and patient nationality on several aspects of the acute diverticulitis course and outcomes among the diverse ethnic populations in Israel.

## 2. Materials and Methods

A retrospective, single-center, cross-sectional study was conducted at the Hillel Yaffe Medical Center, a university-affiliated hospital in Israel. All patients older than 18 years admitted between the years 2006–2018 to the surgery ward who received a discharge diagnosis of either “acute colonic diverticulitis”,” Diverticulitis of Colon with Hemorrhage”, “ Diverticulitis of Colon with Perforation”, “ Diverticulitis with Diverticular Abscess”, “Colonic Diverticular Disease”, with identifying codes of International Classification of Diseases (ICD-9) of 562.11, 562.13, 562.12, 562.10, respectively, were eligible for inclusion in the study. From the large data, we included patients presenting with their first episode of AD in order to follow their disease course and outcomes. Only patients with a CT-confirmed diagnosis of AD were included in the final analysis. Patients with a history of inflammatory bowel disease, colonic cancer or past colonic surgeries were excluded.

We derived relevant data from electronic medical records and discharge reports. Demographic data, including age, sex, ethnicity, and place of birth, were documented upon hospital admission and based on patients’ identification and national databases. Ethnicity was categorized according to the Israeli Central Bureau of Statistics (CBS) into the religious ethnicity of two main groups, Arabs and Jews; we performed a subgroup division of the Jewish population according to their place of birth: Ashkenazi (born in the United States, Europe or former USSR), Sephardic (born in Asia, the Middle East or North Africa) and native Israeli (for whom both parents were born in Israel).

Relevant clinical data, including date of admission, length of admission, colonic distribution of diverticulitis according to CT scan, complications and recurrent episodes, were also incorporated. Complications were categorized according to Hinchey classifications, including fistula, abscess, or perforation. After patients’ discharge from index hospitalization, we tracked relevant reports from clinic, hospital, or primary care visits to identify disease course and the rate of recurrent episodes of acute diverticulitis.

First, we compared young patients presenting with their first episode at age <50 to those aged 50 years or older, in terms of clinical and hospitalization records, as well as clinical course and outcome. Second, demographic and clinical characteristics including age at presentation, complicated diverticulitis rate, hospitalization length, recurrent episodes’ rates between the major ethnic groups, and Jewish and Arab populations were compared. Third, we performed a sub-group analysis in the Jewish population to compare demographic and clinical findings among the various Jewish ethnic subgroups and evaluate their effect on disease outcomes. We performed a multivariate analysis to identify independent factors associated with complicated disease.

### Statistical Analysis

Continuous parameters were presented by means ± standard deviations, and categorical parameters were expressed using frequencies and percentages. Differences between the age and ethnicity groups were compared by *t*-test in quantitative parameters, and Fisher’s exact test in the categorical parameters. Multivariate analysis by logistic regression was used for prediction of complicated course, adjusting for age, gender, ethnic group, place of birth and first vs. recurrent episode. *p* < 0.05 was considered as significant. SPSS version 25 was used for the all statistical analyses. 

## 3. Results

Overall, 637 patients were hospitalized with a radiologically confirmed first episode of diverticulitis between the years 2006–2018. The patient’s age ranged from 19 to 97, with a mean age of 58.2 ± 14.1 years. A slight female predominance was noted (55%). The Jewish population comprised the majority of the total cohort 540 (85%); among them, 218 (40%) were classified as Ashkenazi, 221 (41%) as Sephardic, 55 (11%) native Israeli, while 46 (8%) were of mixed origins. Only 95 patients (15%) were Arabs, reflecting their percentage as a minority population in Israel. When classified by country/place of birth, 348 (54.6%) patients were born in Israel, while the others immigrated from different geographical regions around the world. 

According to the CT scan performed upon admission, more than 94% of diverticulosis was located in the sigma and distal colon. A complicated diverticulitis course was documented in 95 (15%) patients; among them, perforation was recorded as the most frequently reported complication. The baseline characteristics of the entire cohort are outlined in Table 1. 

In the age group analysis, the young age (<50) group had significantly higher rates of native Israeli (85% vs. 43.8%, *p* < 0.01) and Arab minority patients (26.9% vs. 11.2%, *p* < 0.01), as well as a male predominance (69.7% vs. 35.8%, *p* < 0.01). These groups did not differ in terms of the outcome as the rates of recurrent episodes (35.3% vs. 37.3%, *p* = 0.19), hospitalization duration (5 ± 4.7 vs. 6 ± 3.2, *p* = 0.09), and complications (17.3% vs. 13.7%; *p* = 0.16) were similar for both age groups (Table 2).

Examining the correlation between religious ethnicity and diverticulitis, delineated in Table 3, revealed that Arabs developed a first episode of diverticulitis at a significantly younger age compared to Jews (51.8 ± 13.9 vs. 59.4 ± 13.8; *p* < 0.01). The percentage of patients presenting at an age younger than 50 years was substantially higher in the Arab population group. Moreover, female predominance was clearly evident in the Jewish population compared to Arabs (57.4% vs. 43.2%; *p* < 0.01). A distal colonic distribution of diverticulitis was equally evident in both groups. Interestingly, the clinical course of diverticulitis was comparable, as neither the hospitalization length (4 (3–6) vs. 4 (3–6); *p* = 0.36) nor the complication rates (15 (16%) vs. 80 (15%); *p* = 0.88) differed between the groups. 

The classification of patients according to their origin/place of birth is provided in Table 4. Patients born in Israel presented with the first episode of diverticulitis at a significantly younger age than patients born abroad (52.0 ± 12.3 (21–89) vs. 65.6 ± 11.3 (19–97); *p* < 0.01) and were predominantly male (56% vs. 30.8%; *p* < 0.01). Recurrent diverticulitis rates were equal for all origins. Patients born in Asia/Middle East (8.5% vs. 17.8%; *p* = 0.09) and North Africa (7.4% vs. 17.8%; *p* = 0.07) had a noticeably decreased rate of complications compared to those born in Israel, but did not reach a statistical significance. Moreover, the Jewish subgroup investigation revealed that, despite the differences in age and gender at presentation between Ashkenazi and Sephardic groups, as shown in Table 5, complications (39.4% vs. 37.6%; *p* = 0.69) as well as recurrent diverticulitis rates (14.7% vs. 16.7%; *p* = 0.6) were comparable.

Altogether, in the univariate analysis, a complicated disease course was more evident in younger patients (58.2 ± 14.1 vs. 71.5 ± 2.1; *p* = 0.01) and was significantly associated with an increased risk for recurrent episodes of diverticulitis (49.5% vs. 35%; *p* < 0.01) (Table 6). Patients with recurrent diverticulitis episodes were likely to be younger compared with those who did not develop recurrent episodes (59.8 ± 14.1 vs. 57.3 ± 13.9; *p* = 0.034). However, other factors, including gender, place of birth, or religious group, were not associated with recurrent episodes (Table 7).

In the multivariate analysis generated to determine possible predictors of a complicated diverticulitis course, neither age nor ethnicity and place of birth were significantly associated with an increased rate of complicated diseases (Table 8). 

## 4. Discussion

The current study aimed to identify predictors for AD course and outcomes, focusing on age and ethnicity as leading prognostic factors, with a possible direct impact on the management of patients.

The influence of ethnicity on the incidence, course and outcomes of different gastrointestinal disorders is well established. Diverticular disease, among the most encountered disorders, particularly in the Western population, attracted great interest in this regard [17]. In recent large-population case–control studies by our group, we demonstrated that diverticulosis was not associated with an increased risk of colorectal cancer and that the rate of advanced neoplasia diagnosis after acute diverticulitis did not differ from that of average risk population [18]. However, little is known about the effect of ethnicity in terms of diverticular disease course and outcomes. Moreover, only scarce data regarding disease epidemiology and course among the different ethnic groups in Israel were published in recent decades. Our center is a community hospital in the central coastal region of Israel, which serves a population of almost 400,000 citizens who reflect the unique diversity of the region’s population: major and minor ethnic populations, urban and rural areas, as well as natives and immigrants.

In the current study, we demonstrated a predominant distal colon involvement of diverticulitis in the overall cohort and within the various ethnic subgroups, including those who emigrated from Asian and African countries. This was in accordance with the well-established distal involvement of diverticular disease reported in Western populations [19], supporting the ongoing modernization and Westernization of Israeli society as a whole. In addition, we demonstrated that Israelis tend to develop the first episode of diverticulitis at a relatively younger age, compared to the figures published in previous reports from Israel and the Western world. This is also in agreement with other observations showing age shifting toward the development of acute diverticulitis in younger patients [20,21,22]. One of the main findings of our study was the significantly younger average age of the minority Arab patients at the time of presentation compared with their Jewish counterparts (51.8 ± 13.9 vs. 59.4 ± 13.8; *p* < 0.01). Of note, almost half of these patients presented with their first episode at ages younger than 50 years, one of the highest rates when compared to those reported in the literature for other various ethnicities [23]. These interesting findings match with a previous Israeli study conducted by Ghersin et al. [24], in which Arabs tended to develop diverticulitis at a significantly younger age than Jews. Genetic, nutritional, microbiological, behavioral, and environmental factors may account for these findings. Moreover, until recently, the Arab population were characterized by a higher birth rate. Our findings, therefore, could also be a reflection of the different age distribution between the Arab and the Jewish populations. The Arab minority has undergone a major transformation during the last decade. Even though they share similar geographical areas with Jews, Arabs differ in socio-economic status, cultural, and dietary characteristics [25]. The Arab population in Israel tends to have, for the most part, a lower socio-economic status and a higher prevalence of cigarette smoking, as well as obesity [26]. Whether these factors contributed to the prominently younger age at presentation is unknown, and further study to investigate their effects is warranted. 

In terms of the colonic distribution of diverticulitis, clinical course, hospitalization length, complication rates, or recurrent episodes, we could not find significant differences between the Arab and Jewish groups. On the one hand, the Arab minority population who attend our hospital are still living in rural areas more than in urban areas, which is considered as a protective factor. On the other hand, this may be offset by the rapid modernization and adoption of Western dietary patterns and lifestyle, as well as the increased rate of obesity and smoking, two reported risk factors associated with a complicated disease course [27]. Moreover, as outlined earlier, a considerable percentage of Arab patients presented at younger ages compared to Jewish patients, which may have an impact on the final outcome. Notably, Israel has a universal, compulsory and equal healthcare system for all ethnic groups. Moreover, patients from all groups were hospitalized in similar surgery wards and received standard care. They were also linked directly to similar local and regional clinics for medical follow-up. Therefore, differences in the insurance status between patients are negligible and the possible effect of healthcare utilization among ethnic groups is thus believed to be minor. 

A similar trend was also noted within the different Jewish subgroups. Although they differed by gender and age at presentation, as outlined earlier, they were characterized by a relatively similar disease course. It was interesting to show a possible lower risk for complications and a recurrence among the patients who originated in the Middle East and North Africa, but this was close to statistical significance (Table 3 and Table 5). To the best of our knowledge, our study is the first to investigate the contribution of different countries of birth on diverticulitis patients’ outcomes. Yet, these results must be considered in perspective, as there is a need to account for and integrate other possible risk factors for a complicated disease course, preferably in prospective studies.

One of the main aims of the current study was to investigate the outcome of patients presenting with a first episode of AD at a young age (50>). Indeed, the changing pattern of the disease in young patients was clearly demonstrated by recent epidemiological studies. However, the natural history and management of AD in this age group are still a matter of debate [28]. Our findings indicate that a younger age at first presentation with AD was not associated with a more severe clinical course or higher recurrence rate. Indeed, in the multivariate analysis, neither young age at presentation nor specific ethnicity were predictors of a complicated AD course. These findings are in line with recent reports showing that AD is not more aggressive in the young patients when compared to the older ones [29,30,31].

The strengths of the current study include the ethnic diversity of the study population and the detailed information on demographics, disease course and patients’ outcomes. Limitations of the study are inherent in its retrospective and single-center design. Repeated studies in other regions of Israel are warranted to establish the generalizability of our findings. A selection bias is possible due to the study design excluding milder cases of acute diverticulitis. Moreover, smoking habits, dietary factors, obesity, NSAIDs and steroids use, as well as other possible risk factors for diverticulitis were not documented, and thus were not included in the final analysis.

## 5. Conclusions

We showed that diverticulitis among the Israeli population is similar to that in Western populations and involves a younger population than in the past, matching with the fact that Israel is an industrial country with a Western lifestyle, despite the heterogeneity of its population. A young age at presentation was not associated with worse outcomes, and patients from different Israeli ethnic groups had relatively similar disease courses and outcomes. 

## Figures and Tables

**Table 1:** Baseline characteristics of patients on admission.

Total of 637 Patients with Radiologically Confirmed Acute Diverticulitis	
Average age (years)	58.2 ± 14.1 (19–97)
Age 50>	167 (26.2%)
Age 50≤	470 (73.8%)
Female gender (%)	352 (55%)
Ethnicity and religions *n* (%)	
Jews	540 (85%)
-Ashkenazi	218 (40%)
-Spanish	221 (41%)
- Native Israeli	55 (11%)
-Unknown	46 (8%)
Arabs	97 (15%)
Diverticulitis location *n* (%)	
Sigmoid colon	325 (51%)
Sigmoid and descending colon	253 (40%)
Descending colon	25 (4%)
Transverse colon	2 (0.03%)
Right colon	32 (5%)
Complications *n* (%)	
Overall	95 (15%)
Fistula	2 (0.3%)
Abscesses	33 (5%)
Perforation	80 (13%)
Obstruction	12 (2%)

**Table 2:** Baseline characteristics and patients’ outcomes in the young (50>) and old (50≤) age groups.

Age Group	≥50 Years (*n* = 470)	<50 Years (*n* = 167)	*p* Value *
Female gender (%)	302 (64.2%)	49 (29.3%)	*p* < 0.001
Birth (Israel)	206 (43.8%)	142 (85%)	*p* < 0.001
Ethnicity (Arabs)	52 (11.2%)	45 (26.9%)	*p* < 0.001
Hospitalization duration (d)	6 ± 3.52	5 ± 4.75	*p* = 0.08
Recurrent episodes (%)	178 (37.3%)	59 (35.3%)	*p* = 0.19
Complications (%)	63 (13.4%)	29 (17.3%)	*p* = 0.12

* *p* value in bold denotes a statistically significant difference (*p* < 0.05).

**Table 3:** Comparison between Jewish and Arab ethnic populations at first acute diverticulitis episodes.

Ethnic Group	Arabs (97)	Jews (540)	*p* Value *
Age	51.8 ± 13.9 (21–85)	59.4 ± 13.8 (19–97)	
Age 50>	45 (46.4%)	122 (22.6%)	<0.001
Age 50≤	52 (53.6%)	418 (77.4%)	<0.001
Gender (female)	42 (43.2%)	310 (57.4%)	<0.001
Distal colon location (%)	88 (91%)	515 (95%)	0.08
First admission length (days)	4 (3–6)	4 (3–6)	0.43
Relapsing episode *n* (%)	32 (33%)	205 (38%)	0.36
Complications *n* (%)	15 (16%)	80 (15%)	0.88

* *p* value in bold denotes a statistically significant difference (*p* < 0.05).

**Table 4:** Acute diverticulitis course and patients’ characteristics according to place of birth, compared to those born in Israel.

Hospital Stay (Days)	Recurrence (%)	Complications (%)	Gender (F)	Age Mean ± SD (Range)	Birth Place (Region)
4 (3–5)	122 (35%)	62 (17.8%)	152 (44%)	52.0 ± 12.3 (21–89)	Israel (*n* = 348)
5 (3–8)	21 (42.0%)	9 (18.0%)	36 (72%)	72.3 ± 10.5 (41–90)	Europe (*n* = 50)
4 (4,5)	5 (33%)	3 (20%)	7 (47%)	59.3 ± 8.9 (41–72)	USA (*n* = 15)
4 (3–6)	52 (42.3%)	14 (11.4%)	85 (69%)	62.6 ± 14.0 (19–90)	USSR (*n* = 123)
4.5 (2–8)	19 (40.4%)	4 (8.5%)	32 (68%)	68.5 ± 10.7 (30–97)	Middle East (*n* = 47)
5 (3–6)	18 (33.3%)	4 (7.4%)	40 (74%)	65.7 ± 8.6 (51–87)	North Africa (*n* = 54)
**P^1^ = 0.018**	P^1^ = 0.35	P^1^ = 1.00	**P^1^ < 0.001**	**P^1^ < 0.001**	*p*-value *^#^
P^2^ = 0.71	P^2^ = 1.00	P^2^ = 0.74	P^2^ = 1.00	**P^2^ = 0.024**	
P^3^ = 0.19	P^3^ = 0.16	P^3^ = 0.16	**P^3^ < 0.001**	**P^3^ < 0.001**	
P^4^ = 0.09	P^4^ = 0.17	P^4^ = 0.09	**P^4^ = 0.004**	**P^4^ < 0.001**	
P^5^ = 0.051	P^5^ = 0.88	P^5^ = 0.07	**P^5^ < 0.001**	**P^5^ < 0.001**	

* *p* value in bold denotes a statistically significant difference (*p* < 0.05). ^#^ P^1,2,3,4,5^ denotes to statistical comparison between those born in Israel to immigrants from Europe (P^1^), USA (P^2^), USSR (P^3^), Middle East (P^4^) or North Africa (P^5^); respectively.

**Table 5:** Diverticulitis course and characteristics in Jewish subgroups.

Jewish Subgroup	Ashkenazi (*n* = 218)	Sephardic (*n* = 221)	*p* Value *
Age (years)	64.4 ± 13.7	59.03 ± 11.6	***p* < 0.0001**
Female gender (%)	146 (67%)	123 (56%)	***p* = 0.019**
Hospitalization duration (days)	4 (3–6)	4 (3–6)	*p* = 0.18
Recurrent episodes (%)	86 (39.4%)	83 (37.6%)	*p* = 0.69
Complications (%)	32 (14.7%)	37 (16.7%)	*p* = 0.60

* *p* value in bold denotes a statistically significant difference (*p* < 0.05).

**Table 6:** Complicated vs. non-complicated acute diverticulitis; a univariate analysis.

Diverticulitis Course	Uncomplicated (*n* = 542)	Complicated (*n* = 95)	*p* Value *
Age	71.5 ± 2.1	61.2 ± 14.1	**0.001**
Gender female	305 (56%)	47 (49.5%)	0.22
Place of birth:			
- Israel	286 (53%)	62 (65%)	0.06
- North Africa	50 (9%)	4 (4%)	0.11
- Europe	41 (8%)	(10%)	0.58
- Russia	110 (20%)	13 (14%)	0.16
- United States	12 (2%)	3 (3%)	0.48
- Middle East/Asia	42 (7%)	4 (4%)	0.19
Religion (Jewish)	460 (85%)	80 (84%)	0.87
First vs. recurrent	237 (43.7%)	37 (38.9%)	0.08

* *p* value in bold denotes a statistically significant difference (*p* < 0.05).

**Table 7:** Recurrent diverticulitis episodes. A univariate analysis.

Diverticulitis	Single Episode (*n* = 400)	Recurrent Episodes (*n* = 237)	*p* Value *
Age (years)	59.8 ± 14.1	57.3 ± 13.9	*p* = 0.034
Female Gender (%)	216 (54%)	136 (57%)	*p* = 0.41
Place of birth (%)			
Israel	226 (56.5%)	122 (51.5%)	*p* = 0.25
North of Africa	36 (9%)	18 (7%)	*p* = 0.65
Europe	29 (7%)	21 (9%)	*p* = 0.54
Russia	71 (18%)	52 (22%)	*p* = 0.21
America	10 (2.5%)	5 92%)	*p* = 1.00
Middle east/Asia	28 (7%)	19 (8%)	*p* = 0.24
Ethnicity (Jewish)	335 (84%)	205 (87%)	*p* = 0.36

* *p* value in bold denotes a statistically significant difference (*p* < 0.05).

**Table 8:** Predictors of a complicated diverticulitis course; a multivariate analysis.

Variable	*p*-Value	Odds Ratio	95% C.I. for Odds Ratio	
			Lower	Upper
Age at test (50y+)	0.19	0.94	0.79	1.09
Gender (female)	0.594	1.134	0.714	1.801
Ethnic group (Arabs)	0.491	1.249	0.663	2.354
Birth place (Israel)	0.091	1.593	0.929	2.730
First vs. recurrent	0.08	1.027	.981	1.075

## Data Availability

The datasets used and/or analyzed during the current study are available from the corresponding author on reasonable request.
